# Caregiver-Provider Communication About Pain in Persons With Dementia

**DOI:** 10.1093/geroni/igaa057.233

**Published:** 2020-12-16

**Authors:** Catherine Riffin, Sylvia Lee, M Cary Reid, Keela Herr, Karl Pillemer

**Affiliations:** 1 Weill Cornell Medical College, London, England, United Kingdom; 2 Weill Cornell Medicine, New York, New York, United States; 3 Weill Cornell Medical College, New York City, New York, United States; 4 University of Iowa, Iowa City, Iowa, United States; 5 Cornell University, Ithaca, New York, United States

## Abstract

Pain in older persons with dementia (PWD) is severely under-detected and under-managed. Family caregivers can play an important role in addressing these disparities by acquiring the requisite skills to communicate PWD’s pain symptoms and behaviors to health care providers, but little is known about how caregivers of dementia patients and their providers approach such pain-related discussions. We employed qualitative methods to explore the perspectives of family caregivers of PWD (n=18) and health care providers (geriatricians, general internists, neurologists, emergency room physicians) involved in PWD’s treatment (n=16) regarding pain communication. We specifically focused on participants’: 1) priorities and expectations for communicating about pain and dementia, 2) challenges to communicating about pain and dementia, and 3) strategies and recommendations for optimizing communication about pain and dementia. Analyses revealed that caregivers and health care providers expected to receive accurate, detailed information from one another, but uncertainty in both groups around differentiating pain behaviors from dementia symptoms acted as a barrier to effective information exchange. Additional challenges to productive pain-related discussions were identified by caregivers, including provider fatalism and lack of interpersonal skills, and by providers, including patient-caregiver disagreement about pain symptoms and unreliable caregiver reporting. Participants endorsed using practical approaches, such as pain scales and logs, as well as rapport-building strategies, such as affirmation of caregivers’ input, to facilitate collaborative discussions.

